# Single-Cell RNA Sequencing Reveals Extensive Heterogeneity and Unique Gene Trajectories in Non-Transformed and Transformed Human Lung Epithelial Cells: Insights into the Role of LncRNAs in Tumor Heterogeneity

**DOI:** 10.3390/ijms26041690

**Published:** 2025-02-16

**Authors:** Sokviseth Moeng, Andres D. Chamorro-Parejo, Minsun S. Jeon, James J. Cai, Kenneth S. Ramos

**Affiliations:** 1 Center for Genomic and Precision Medicine, Texas A&M Institute of Biosciences and Technology, Texas Medical Center, Houston, TX 77030, USA; sokvisethmoeng@tamu.edu (S.M.); achamorro@tamu.edu (A.D.C.-P.); 2 Center for Epigenetics and Disease Prevention, Texas A&M Institute of Biosciences and Technology, Texas Medical Center, Houston, TX 77030, USA; minsunjeon@tamu.edu; 3 Department of Veterinary Integrative Biosciences, Texas A&M University, College Station, TX 77843, USA; jcai@tamu.edu

**Keywords:** lung cancer, single-cell RNA sequencing, protein-coding genes, cellular heterogeneity, carcinogen, benzo(a)pyrene, lncRNAs

## Abstract

Lung cancer exhibits substantial inter- and intra-tumor heterogeneity, with features that present significant challenges in advancing biomarker discovery and the development of targeted therapeutics. To fill this gap, we employed single-cell RNA sequencing (scRNA-seq) and advanced bioinformatics tools to evaluate the transcriptomic heterogeneity of immortalized, non-transformed (BEAS2B) and transformed (H460) lung epithelial cell lines and their responses to carcinogen challenge. Gene expression profiles resolved four primary clusters further discretized into unique subclusters based on genetic signatures and phenotypic profiles. Profiles of long non-coding RNAs (lncRNAs) identified microRNA host genes, antisense RNA genes, divergent transcript, and long intergenic non-coding RNAs as contributors to cellular heterogeneity. These findings indicate that distinct patterns of gene expression, remarkably in lncRNAs, define cellular heterogeneity in non-transformed versus transformed cells. These features can be exploited for the development of therapies directed at specific cell subpopulations in precancerous lesions and within lung tumors.

## 1. Introduction

With an estimated 2.2 million new cases worldwide and 1.79 million deaths annually, lung cancer remains the leading cause of cancer-related mortality in the United States and worldwide [1]. For non-small-cell lung cancer (NSCLC) patients diagnosed at advanced stages, the prognosis is poor, with nearly half succumbing to the disease within the first year due to the limited efficacy of treatments during the late stages of disease [2,3]. Lung cancer cell heterogeneity at both the cellular and histological levels contribute significantly to deficits in the accurate diagnosis and treatment of the disease [4,5]. Bischoff et al. have emphasized the critical role of cellular heterogeneity in defining distinct microenvironmental patterns for prognostic evaluation [6].

LncRNAs, a group of non-coding transcripts longer than 200 nucleotides, are extensively transcribed throughout the genome and are known to interact with DNA, RNA, and proteins [7,8]. Their role in tumor heterogeneity remains largely unexplored, particularly in the context of investigations using single-cell RNA sequencing (scRNA-seq) technologies. Liang et al. introduced an immune-associated lncRNAs signature as a biomarker of tumor immune heterogeneity in advanced nasopharyngeal carcinoma [9]. Zhao et al. characterized the inter-tumor heterogeneity of differentially expressed lncRNAs in breast cancer [10]. scRNA-seq was used by Li et al. to profile the heterogeneity of lncRNA expression in renal cell carcinoma [11]. We posit that unraveling lncRNA expression profiles and their regulatory networks in non-transformed or transformed cells can inform the classification of cancer subtypes as cells transition toward malignant states and identify diagnostic and prognostic biomarkers.

In this study, we utilized scRNA-seq to characterize protein-coding and non-coding (mostly lncRNA) gene expression heterogeneity in immortalized, non-transformed BEAS2B cells compared to H460 lung cancer cells. Given the importance of environmental injury in lung cancer onset and progression, we also examined the heterogeneity of response to carcinogen challenge. Significant differences in protein-coding and non-coding genes were identified, with BEAS2B cells exhibiting considerable degrees of heterogeneity in gene expression compared to H460 cells under both basal and carcinogen-stimulated conditions. Distinctive patterns of gene expression, especially in lncRNAs in precancerous and cancerous cells, can be exploited to develop targeted therapies directed at specific liabilities within dominant cell subpopulations.

## 2. Results

### 2.1. Comparative Gene Expression Profiles Between BEAS2B and H460 Lung Epithelial Cell Lines

**Figure 1:**
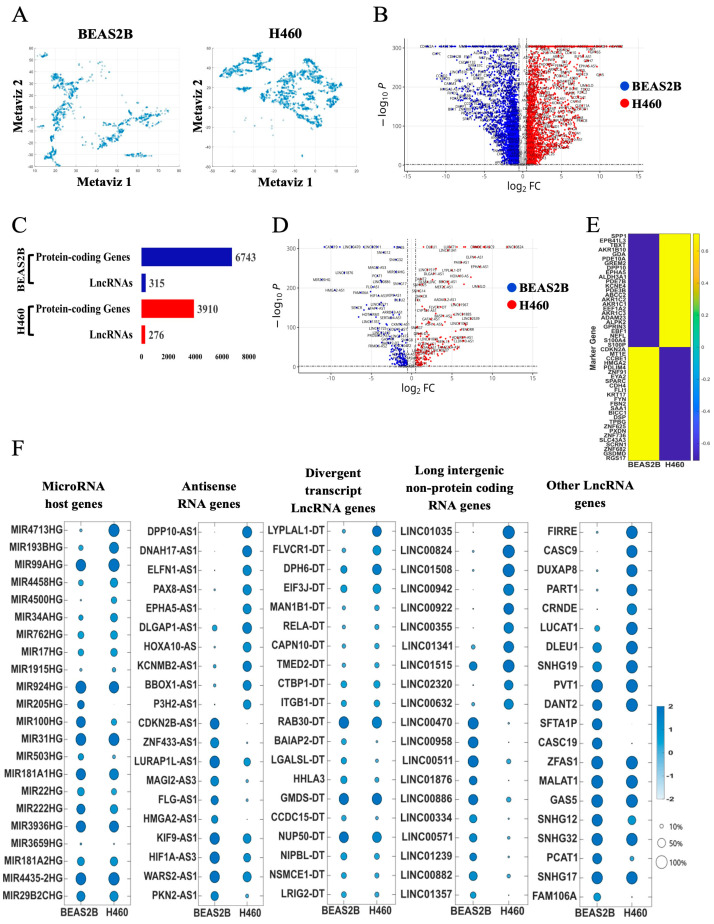
Differential gene expression profiles in non-transformed BEAS2B and transformed H460 lung epithelial cell lines. (**A**) Metaviz projection of BEAS2B and H460 single cells. (**B**) Volcano plot of all DEGs. Blue denotes DEGs upregulated in BEAS2B cells and downregulated in H460 cells, while red denotes DEGs upregulated in H460 cells and downregulated in BEAS2B cells. Gray indicates non-significant changes. (**C**) Bar plot of the number of DEGs in BEAS2B and H460 cells. Color coding as described above, with blue comparing DEGs in BEAS2B to H460 cells and red comparing DEGs in H460 cells to BEAS2B cells. Genes were classified into protein-coding versus non-coding RNA genes (mostly lncRNAs). (**D**) Volcano plots comparing lncRNA genes in BEAS2B to H460 cells. Color coding as described above. (**E**) Heatmaps displaying expression patterns of the top 50 protein-coding genes (row) in BEAS2B and H460 cells in (**B**). (**F**) Dot plots showing top lncRNAs in BEAS2B and H460 cells in (**D**). LncRNA types included microRNA host genes, antisense RNA genes, divergent transcript lncRNA genes, long intergenic non-protein-coding RNA genes, and other lncRNA genes. Gene expression levels are defined by color intensity (light blue = low; dark blue = high). The percentage of cells expressing lncRNAs is indicated by the size of the circle.

**Figure 2:**
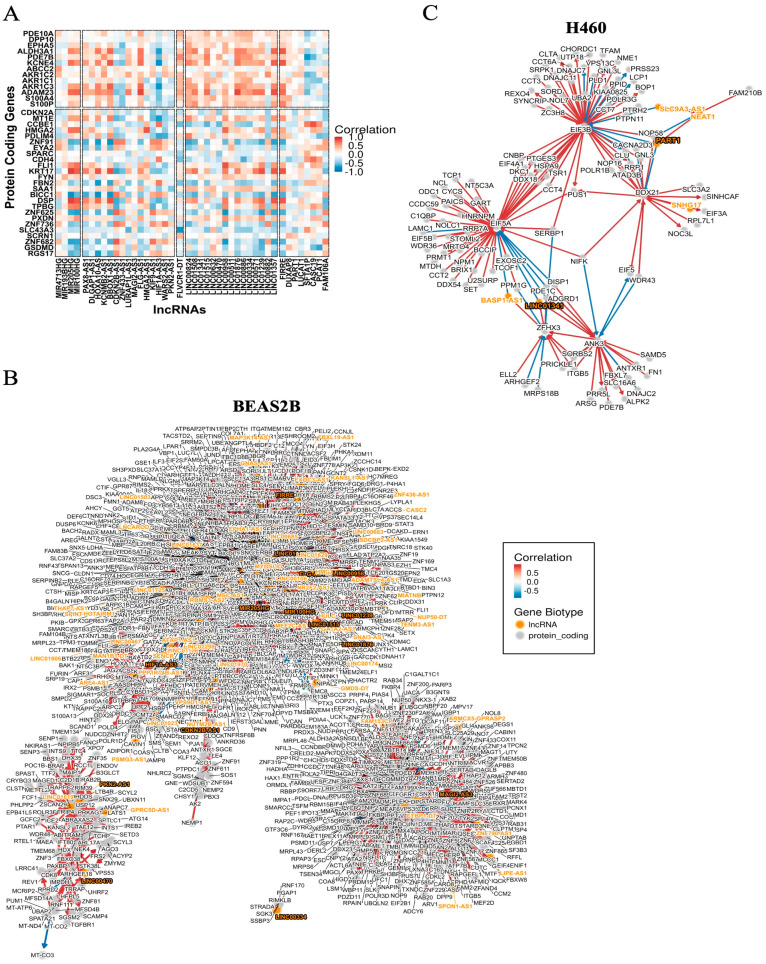
Regulatory network for the top differentially expressed LncRNAs in BEAS2B and H460 cells. (**A**) Correlation matrix plot between the top differentially expressed protein-coding genes and the most varied lncRNAs identified in BEAS2B and H460 cells. Color intensity indicates the degree of correlation between protein-coding and lncRNAs, with red and blue denoting positive and negative correlations, respectively. (**B**,**C**) The lncRNA regulatory networks in the BEAS2B or H460 cell lines, respectively, illustrate the regulatory relationships among lncRNA and protein-coding genes in BEAS2B or H460 cells. Orange denotes lncRNAs and gray denotes protein-coding genes. Color intensity symbolizes the degree of correlation between lncRNA and protein-coding genes, with red representing positive correlation and blue representing negative correlation.

The hierarchical structure and dimensionality of the scRNA-seq datasets are depicted in Figure 1A. A total of 7058 and 4186 differentially expressed genes (DEGs) were identified, including 6743 protein-coding genes and 315 lncRNAs in BEAS2B cells and 3910 protein-coding genes and 276 lncRNAs in H460 cells (Figure 1B–D). The top differentially expressed protein-coding genes in BEAS2B cells were linked to RNA splicing, double-strand break DNA repair, autophagy, and DNA replication, while those in H460 cells were primarily linked to cellular metabolic and mitochondrial pathways (Figure 1E, Figure S1, and Table S1). LncRNAs were differentially expressed in BEAS2B cells compared to H460 cells, with antisense RNAs and long intergenic non-coding RNAs (LincRNAs) showing the most notable differences (Figure 1F and Table S2). A correlation matrix plot of the top differentially expressed protein-coding genes in both cell lines showed strong positive and negative correlations with the top differentially expressed lncRNA targets (Figure 2A). Construction of the regulatory networks for the highly expressed lncRNAs, coupled with co-expression analyses, identified novel associations with protein-coding genes in both cell types (Figure 2B,C and Figure S2). BEAS2B cells exhibited a more complex and heterogenous network of lncRNAs than H460 cells.

### 2.2. Cellular Heterogeneity of BEAS2B Cells and Their Response to Carcinogen Challenge

**Figure 3:**
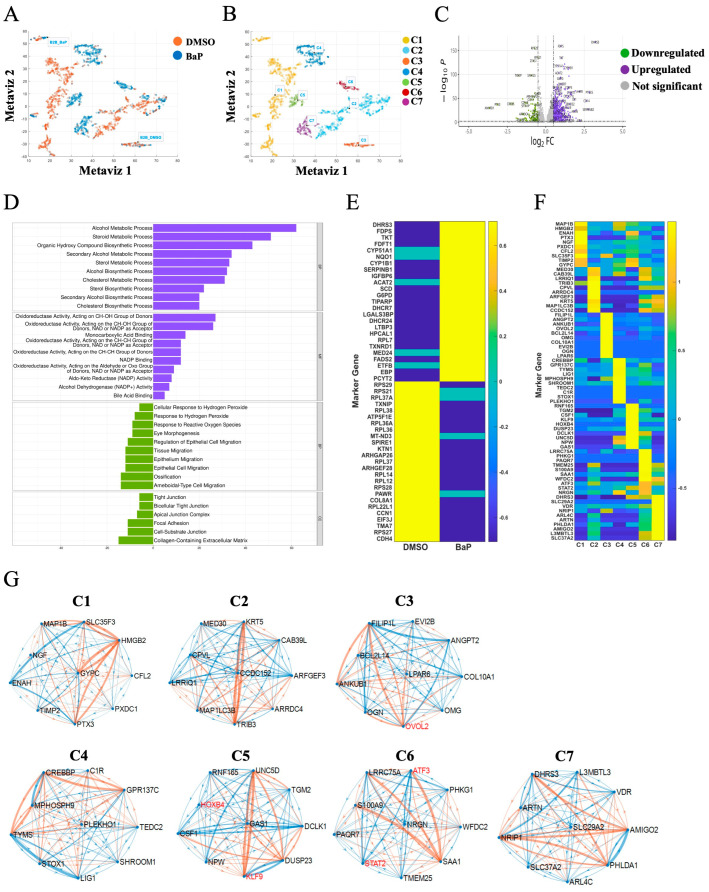
Heterogeneity of non-transformed BEAS2B cells and their response to carcinogen challenge. (**A**) Cell projection of BEAS2B cells treated with DMSO or BaP. (**B**) The main cell clusters identified were stratified into seven subclusters using K-means clustering. Three cell subclusters were identified for BEAS2B-DMSO (C1–C3) and four subclusters for BEAS2B-BaP (C4–C7). (**C**) Volcano plots of DEGs in BEAS2B cells challenged with BaP compared to cells treated with DMSO. Violet denotes upregulated genes, green denotes downregulated genes, and gray denotes non-significant changes in gene expression. (**D**) GO enrichment analysis using three main categories: biological process (BP), cellular component (CC), and molecular function (MF) with an adjusted *p*-value < 0.05. The *x*-axis indicates the number of genes in each category. The top ten terms among these three categories were identified. Violet denotes upregulated genes and green denotes downregulated genes. (**E**) Heatmap illustrating the top 50 differentially expressed protein-coding genes following BaP treatment. (**F**) Heatmap showing a set of the top ten genes expressed in each subcluster compared to each other and representing subcluster-specific gene signatures. (**G**) GRN analysis of subcluster-specific gene signatures. The blue link denotes positive correlations between two genes and the orange link denotes negative correlations between two genes. The weight of the line indicates the strength of the correlation, and the red highlight identifies known transcription factors.

We next examined the profiles of BEAS2B cells under control (DMSO) and Benzo(a)pyrene (BaP)-treated conditions (Figure 3A). K-means clustering identified three subclusters in control cells (C1–C3) and four subclusters in BaP-treated cells (C4–C7) (Figure 3B). These clusters were further stratified into up- and downregulated genes, with 1097 and 362 genes upregulated and downregulated, respectively (Figure 3C). The number of cells in each cluster is presented in Table S3.

GO enrichment analysis identified neurogenesis, apoptosis, and DNA binding as dominant pathways in C1, ubiquitin-protein transferase activity in C2, and collagen-containing extracellular matrix and extracellular matrix structure as weak signals in C3 (Figure S3). No prominent signatures were found in control cells for downregulated genes. Carcinogen treatment upregulated pathways involved in alcohol and steroid metabolism, organic hydroxy compound biosynthesis, and sterol and cholesterol metabolism, and it downregulated ossification and tissue and epithelial cell migration (Figure 3D). Weak signatures were found for signaling/nuclei and morphogenesis in C4 and C5, respectively, and phosphate ion transport in C7 (Figure S3). The top 50 DEGs in DMSO- and BaP-treated BEAS2B cells are depicted in Figure 3E and listed in Table S4, with signatures for the top 10 DEGs in each subcluster are presented in Figure 3F. Gene regulatory network (GRN) analyses identified OVOL2 in C3, HOXB4 and KLF9 in C5, and ATF3 and STAT2 in C6 as key transcription factors in BEAS2B cells (Figure 3G).

### 2.3. Cellular Heterogeneity of H460 Cells and Their Response to Carcinogen Challenge

**Figure 4:**
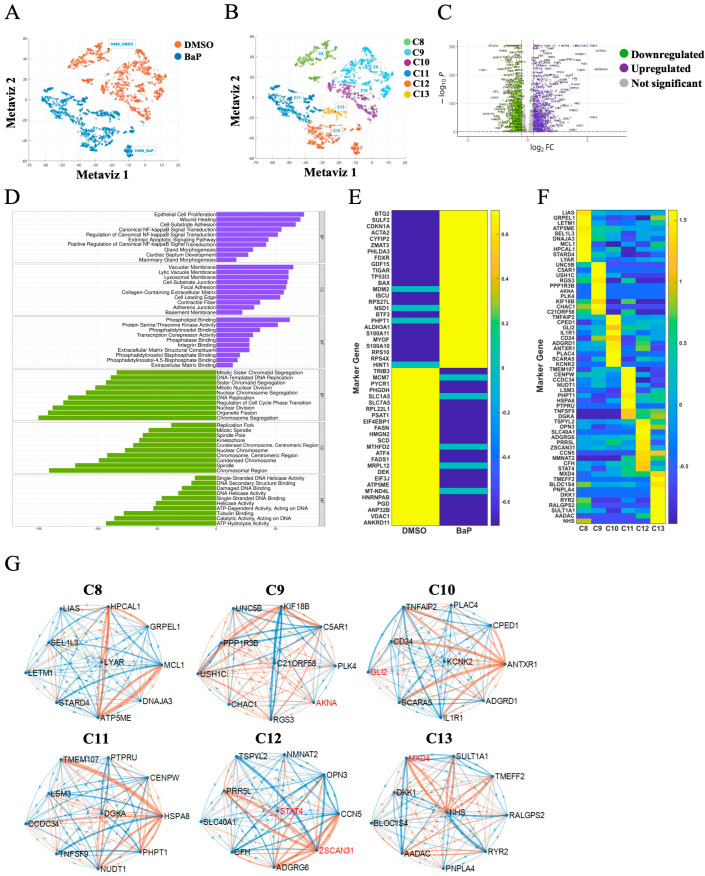
Heterogeneity of transformed H460 cells and their response to carcinogen challenge. (**A**) Cell projection of H460 cells treated with DMSO or BaP. (**B**) The main cell clusters identified were further stratified into six subclusters using K-means clustering. Three cell subclusters each were identified for H460-DMSO (C8–C10) and H460-BaP (C11–C13). (**C**) Volcano plots of DEGs in H460 cells treated with BaP compared to DMSO. Violet denotes upregulated genes, green denotes downregulated genes, and gray denotes non-significant changes. (**D**) GO enrichment analysis using three main categories: biological process (BP), cellular component (CC), and molecular function (MF) with an adjusted *p*-value < 0.05. The *x*-axis indicates the number of genes in each category. The top ten terms of these three categories were identified. Violet denotes the GO enrichment for upregulated genes, while green denotes downregulated genes. (**E**) Heatmap illustrating the top 50 differentially expressed protein-coding genes following BaP treatment. (**F**) Heatmap showing a set of the top ten genes in each subcluster, compared to each other and representing subcluster-specific gene signatures. (**G**) GRN analysis of subcluster-specific gene signatures. The blue link denotes positive correlations between two genes and the orange link denotes negative correlations between two genes. The weight of the line indicates the strength of the correlation, and the red highlight identifies known as transcription factors.

The differences between DMSO- and carcinogen-treated H460 cells are depicted in Figure 4A, with C8–10 identified as subclusters in DMSO-treated cells and C11–C13 as subclusters of BaP-treated cells (Figure 4B). The number of cells in each cluster is presented in Table S3. Upregulated and downregulated genes are shown in Figure 4C, with 1626 upregulated genes and 1981 downregulated genes, respectively. GO enrichment analysis identified epithelial cell proliferation, wound healing, cell–substrate adhesion, NF-kB signaling, extrinsic apoptotic signaling, and inflammation as upregulated pathways and chromosome segregation, organelle fission, nuclear division, regulation of cell cycle phase transition, DNA replication, and cell cycle regulation as downregulated pathways (Figure 4D). The top 50 DEGs are presented in Figure 4E and listed in Table S5. Specific subcluster signatures are displayed in Figure 4F, with GO enrichment analysis for each subcluster summarized in Figure S3. The predominant pathways in control H460 cells included protein and proton transmembrane transport and protein folding in C8 and cytokine stimulus and cytokine-mediated signaling in C10. In contrast, the predominant pathways for BaP-treated H460 cells included trabecula formation and morphogenesis in C12, and triglyceride catabolism, neutral lipid catabolism, and acylglycerol catabolism in C13. Weaker signals were identified in C9 and C11, including development and inflammasome/chromatin, respectively. Signatures for downregulated genes showed no notable differences among the subclusters. Unique transcription factor networks identified in specific subclusters included AKNA in C9, GLI2 in C10, STAT4 and ZSCAN31 in C12, and MXD4 in C13 (Figure 4G). GO enrichment analysis of DEGs in carcinogen-treated BEAS2B and H460 cells identified DNA repair and DNA replication as the dominant processes in BEAS2B cells compared to signaling, developmental growth, and metabolism in H460 cells (Figure S4).

### 2.4. LncRNA Profiles in BEAS2B and H460 Cells Under Basal and Carcinogen-Stimulated Conditions

**Figure 5:**
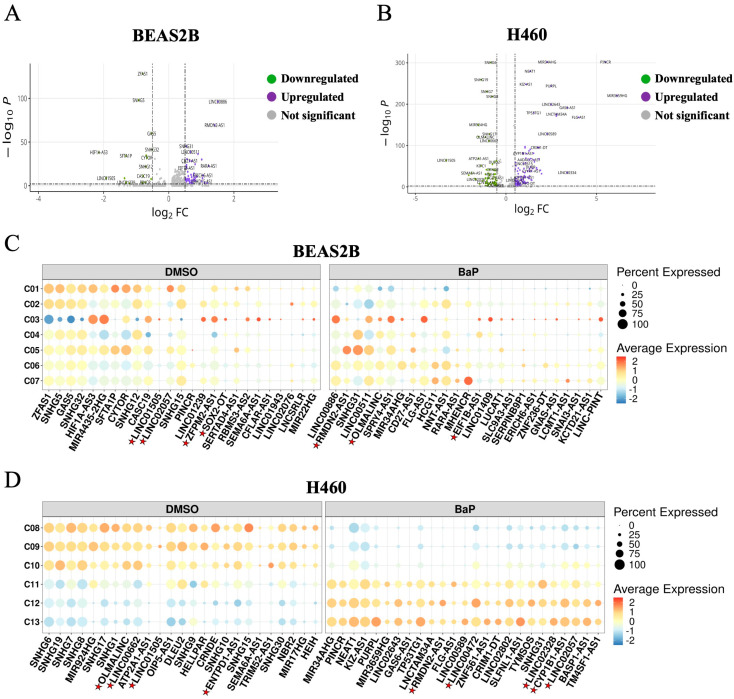
Identification of LncRNA gene expression profiles in response to carcinogen challenge. Volcano plots showing differentially expressed lncRNAs in (**A**) BEAS2B cells and (**B**) H460 cells following BaP exposure. Violet denotes upregulated lncRNAs, green denotes downregulated lncRNAs, and gray denotes non-significant changes. (**C**) Dot plot showing the expression profiles of the top 25 lncRNAs in DMSO-treated BEAS2B cells and the top 25 lncRNAs in BaP-treated BEAS2B cells, categorized by their respective subclusters. (**D**) Dot plot displaying the expression profiles of the top 25 lncRNAs in DMSO-treated H460 cells and the top 25 lncRNAs in BaP-treated H460 cells, organized within their respective subclusters. Color intensity denotes the average expression of the gene within a subcluster while the size denotes the percentage of cells expressing the lncRNA. The red star marks LINE-1-embedded lncRNAs.

**Figure 6:**
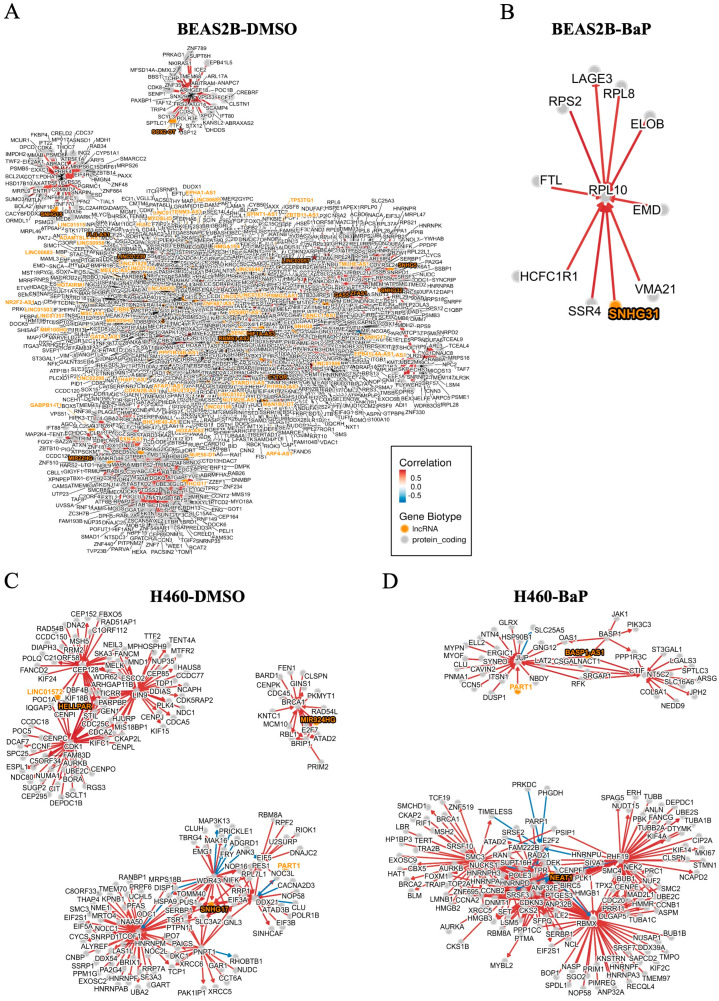
Integrated regulatory network of the top differentially expressed LncRNAs in BEAS2B and H460 cells upon DMSO or carcinogen challenge. (**A**,**B**) Regulatory network of the top differentially expressed lncRNA genes in BEAS2B cells treated with DMSO or BaP, respectively, highlighting regulatory interactions of lncRNAs and protein-coding genes in BEAS2B cells. (**C**,**D**) Regulatory network of the most differentially expressed lncRNAs in DMSO- or BaP-treated H40 cells, emphasizing the regulatory interactions between lncRNAs and protein-coding genes in H460 cells. Orange denotes lncRNA and gray denotes protein-coding genes. Color intensity denotes the degree of correlation between lncRNA and protein-coding genes, with red denotes positive correlation and blue denotes negative correlation. The red star marks LINE-1-embedded lncRNAs.

LncRNAs were the largest class of non-coding RNAs identified in BEAS2B and H460 cells. Upregulated lncRNAs after BaP treatment are shown in Figure 5A and Figure 5B, respectively. Dot plots revealed greater lncRNA heterogeneity in BEAS2B cells compared to H460 cells (Figure 5C,D), a finding consistent with the intricate interplay of lncRNAs and protein-coding genes in BEAS2B cells relative to H460 cells following DMSO and carcinogen treatment (Figure 6A–D and Figure S5). Recent studies have shown that lncRNAs can contain transposable elements (TEs) that impact lncRNA sequence, localization, function, and regulation [12]. This was confirmed in our study with the identification of ancestral and young long interspersed nuclear element-1 (LINE-1) retrotransposons in lncRNAs, marked with a red star symbol in Figure 5C,D and listed in Table S6.

## 3. Discussion

The conventional view of cells as the essential functional units in multicellular organisms often overlooks their inherent diversity in any given tissue [13]. This diversity becomes relevant as cells transition from healthy to disease states in response to external and internal signals, as modeled here by comparing cells under basal and carcinogen-stimulated conditions. In our study, we harnessed the power of scRNA-seq technology to define genetic signatures in immortalized, non-transformed BEAS2B cells compared to transformed H460 lung cancer epithelial cells, as well as their responses to carcinogen challenge. Significant differences were observed in both protein-coding and non-coding genes between the two cell types, with BEAS2B cells exhibiting greater heterogeneity compared to H460 cells. Under basal conditions, albeit following treatment with DMSO, BEAS2B gene expression was associated with genes involved in RNA splicing, double-strand break DNA repair, autophagy, and DNA replication. In contrast, H460 cell profiles involved cellular metabolic pathways and mitochondrial processes. These differences likely reflect the need of non-transformed cells to preserve genomic integrity, while the H460 cells harboring an oncogenic KRAS mutation seek to optimize metabolic efficiency to support aggressive growth and survival.

After carcinogen exposure, BEAS2B cells upregulated pathways related to cellular metabolism and downregulated, migration, remodeling, and structural integrity. H460 cells upregulated epithelial proliferation, wound healing, and cell–substrate adhesion pathways, while cell cycle and mitotic processes were downregulated. These responses are consistent with the known effects of BaP on cell proliferation, DNA damage, cell cycle regulation, invasion, migration, apoptosis [14,15,16], and fibrotic changes that impair differentiation of lung stem cells [17]. A greater number of differentially expressed protein-coding genes was identified in BEAS2B cells relative to H460 cells, a finding consistent with a higher degree of heterogeneity. In addition, significant differences in lncRNA expression impacting antisense RNA genes and long intergenic non-protein-coding RNA genes were seen in both cell types. The global co-expression network of lncRNAs paralleled the heterogeneous features shown by BEAS2B cells, with a more complex interactive network compared to H460 cells. These findings unveiled differences between BEAS2B and H460 cells that span both protein-coding and non-coding regions of the genome.

Subcluster-specific signatures confirmed the extensive heterogeneity of BEAS2B cells, clustering into three distinct groups. C1 was enriched with genes involved in neurogenesis, apoptosis, and DNA binding; C2 with ubiquitin-protein transferase activity, NF-kB, and cytokines; and C3 with collagen-containing extracellular matrix. Carcinogen exposure triggered weak signals in C4 through C6 involving morphogenesis, with changes in phosphate ion transport seen in C7. Discretization of GRNs identified OVOL2, an epithelial lineage determinant in C3 [18]; HOXB4, a regulator of ovarian cancer progression and embryonic stem cell differentiation in C5 [19,20,21]; KLF9, an inhibitor of gastric cancer invasion and androgen-dependent prostate cancer growth in C5 [22,23]; ATF3, a stress-regulated signal in metabolism, immunity, and oncogenesis in C6 [24]; and STAT2, a regulator of proliferation, metastasis, and chemoresistance in C6 [25,26,27,28]. In sharp contrast, H460 cells displayed remarkably stable patterns of gene expression, likely reflecting the clonal selection inherent to established cancer cell lines. The stability of gene expression in H460 cells suggests tighter regulatory control following genetic damage. Of note are the significant changes in metabolic and catabolic processes seen in the C13 subcluster of H460 cells.

The transcribed genome predominantly consists of non-coding RNAs (ncRNAs), with lncRNAs representing the largest category of ncRNAs involved in the regulation gene expression at the transcriptional and post-transcriptional levels [8,29,30,31]. LncRNAs have been implicated in several cancers, including lung cancer, though their functions remain a topic of debate [30,32,33,34]. Under basal conditions, the most distinctive lncRNA variations between BEAS2B and H460 cells involved antisense RNAs and long intergenic non-protein-coding RNAs, with several unique patterns emerging after carcinogen exposure and with BEAS2B cells showing a more heterogenous pattern under both basal and carcinogen-stimulated conditions. Regarding lncRNAs upregulated in BEAS2B cells, CDKN2B-AS1 has been reported to be aberrantly expressed in multiple cancers [35]. MAGI2-AS3 has been shown to exert inhibitory effects on the progression of renal cell carcinoma, bladder cancer, and NSCLC [36,37,38,39]. Liang et al. reported that LINC00958 represses colorectal cell apoptosis and radiosensitivity [40]. Additionally, Ma et al. described the inhibitory effects of LINC00886 on the anaplastic thyroid malignancy [41]. In contrast, DPP10-AS1, a highly upregulated lncRNA in H460 cells compared to BEAS2B cells, is an oncogenic lncRNA that promotes lung cancer malignancy [42]. DNAH17-AS1 has been reported to promote NSCLC and pancreatic tumorigenesis [43,44]. Similarly, ELFN1-AS1 has been implicated in promoting gastric, colorectal, and ovarian cancer growth, migration, invasion, and metastasis [45,46,47]. Regarding upregulated LincRNAs in H460 cells, Xiao et al. reported that LINC01508 is downregulated in cisplatin-resistant ovarian cancer [48]. Wang et al. described the oncogenic role of LINC00942 enhancing lung adenocarcinoma proliferation [49]. Additionally, LINC00922 has been shown to aggravate the malignancies of various types of cancers, including lung cancer [50], colorectal cancer [51], ovarian cancer [52], and gastric cancer [53,54,55]. The tumor-inhibitory functions of lncRNAs highly expressed in BEAS2B cells suggest that their upregulation in non-transformed cells may either exert tumor-suppressive functions or serve to support normal cellular processes such as proliferation and survival in this cell line. This is consistent with our GO enrichment analysis, which highlighted DNA repair and genome integrity as the predominant pathways in BEAS2B cells. On the other hand, the oncogenic functions of lncRNAs highly expressed in transformed H460 cells suggest that these lncRNAs may contribute to enhanced tumorigenesis in lung cancer. However, their exact roles need to be experimentally validated, and future studies will be required to explore these relationships in more detail. Notably, the identification of LINE-1 sequences within lncRNAs in both BEAS2B and H460 cells raises interesting questions about functional interactions between lncRNAs and transposable elements. It remains to be determined whether LINE-1 sequences serve as functional domains within lncRNAs, providing a compelling framework for studying lung cancer heterogeneity and its impact on therapeutic efficacy and disease progression. It should be noted that the measurement of lncRNA gene expression using the 10× Genomics 3′ single-cell gene expression platform carries a poly(A) bias, as it uses poly(dT) primers for reverse transcription [56,57]. While many lncRNAs are polyadenylated and thus detectable [58], those lacking a poly(A) tail may be missed entirely.

Lung cancer is a highly heterogeneous disease, with lung squamous carcinoma exhibiting greater intra-tumor and inter-tumor heterogeneity compared to lung adenocarcinoma [59]. Wu et al. highlighted the inter- and intra-tumor heterogeneity of NSCLC by performing scRNA-seq and mapping the cell type-specific transcriptome landscape of cancer cells and their tumor microenvironment in advanced NSCLC patient samples [60]. Rare cell types in tumor like follicular dendritic cells and T helper 17 cells were identified, and tumors from various patients exhibited significant diversity in their cellular components, chromosomal structure, developmental pathways, and dominant phenotypes [60]. Our study indicated that in addition to the heterogeneity contributed by infiltrating immune cells, intra-tumor heterogeneity is also defined by the variable nature of gene expression, notably lncRNAs in lung epithelial cell subpopulations. Hence, lncRNAs maybe more cell-type specific than protein-coding genes. Such variations may impact tumor behavior and response to treatment. While we did not compare different tumor cell types with differences in mutation load, our comparison of precancerous and cancerous cells identified notable differences that could be exploited to develop targeted therapies directed at specific genes within dominant cell subpopulations.

## 4. Materials and Methods

### 4.1. Cell Lines and Carcinogen Treatment

Cell lines were purchased from the American Type Culture Collection (ATCC, Manassas, VA, USA) and cultured under standard conditions with 5% (*v*/*v*) CO_2_ at 37 °C. The immortalized, non-tumorigenic BEAS2B cell line and the KRAS Q61H transformed NCI-H460 cell line [61] were grown in LHC-9 and RPMI-1640 media, respectively. All cell lines were verified as pathogen- and mycoplasma-free. Benzo(a)pyrene (BaP) (Sigma-Aldrich, St. Louis, MO, USA) was dissolved in dimethyl sulfoxide (DMSO) and added to cultures for 48 h at a final concentration of 1 μM. Control cultures received an equivalent volume of DMSO.

### 4.2. Library Preparation and scRNA-Seq

Single cells were isolated, and cDNA synthesis, barcoding, and sequencing library preparation performed using the 10× Genomics Chromium Next GEM Single Cell 3′ Reagent Kits v3, following the manufacturer’s instructions. The quality of the libraries was confirmed using an Agilent Bioanalyzer (Agilent Technologies, Santa Clara, CA, USA). The scRNA-seq was conducted under basal conditions and after BaP exposure using an Illumina sequencer (Illumina, San Diego, CA, USA), yielding an average of 110,000 reads per cell (Figure S6A). Quality control (QC) steps were implemented to remove low-quality reads.

### 4.3. Quality Control

Fixed thresholds were applied to exclude low-quality cells, minimizing technical noise, and ensuring that only high-quality cells were included in the expression matrix. QC measures included (1) the exclusion of genes expressed in fewer than 10% of total cells, (2) the removal of cells with minimal gene expression, where minimal gene expression denotes values close to zero, and (3) the elimination of cells expressing more than 15% mitochondrial genes (percent_Mito) [62]. Additionally, ribosomal RNA, hemoglobin, and platelet genes were excluded (Figure S6B,C). Normalization was performed after QC and the clean datasets used for further analysis.

### 4.4. Raw scRNA-Seq Data

Data were processed using Cell Ranger (version 7.2.0) and aligned to the human reference genome (GRCh38-2020-A). All libraries achieved over 95% valid barcode reads, with approximately 90% of reads confidently mapped to the reference genome. The Cell Ranger pipeline was used for mapping and quantifying gene expressions. A total of approximately 7500 cells were sequenced, including 1803 BEAS2B-DMSO, 1427 BEAS2B-BaP, 2390 H460-DMSO, and 1842 H460-BaP cells. The average number of cells per sample was approximately 1800 cells (Table S7).

### scRNA-Seq Data Analysis

#### 4.5.1. Clustering Analysis

Dimensionality reduction and clustering were conducted using the meta-visualization method available in Metaviz [63] and K-means clustering algorithms, respectively. These analyses were performed using the scGEAToolbox in Matlab version R2024a [64]. Subclusters within the same treatment group and exhibiting similar gene expression patterns were merged before downstream analysis.

#### 4.5.2. Differentially Expressed Genes (DEGs) Analysis

DEGs were identified using the FindAllMarkers function, with adjusted *p*-values calculated using the Wilcoxon rank-sum test. Heatmaps of DEGs and gene regulatory networks (GRNs) were generated using the scGEAToolbox in Matlab version R2024a [64]. The EnhancedVolcano tool was used to visualize DEGs [65], with threshold values based on log2 fold changes (FC) and −log10(*p*-value). In this study, threshold values used to generate the volcano plot were: log2 FC = 0.5 and −log10(*p*-value) = 2, where *p*-value < 0.01. Dot plots of DEGs were generated in Seurat, with dot size representing the percentage of cells expressing a particular gene, and the shade of dot colors indicating the intensity of gene expression.

#### 4.5.3. Functional Enrichment Analyses

Gene Ontology (GO) enrichment analysis was performed using the Enrich function from the clusterProfiler package [66]. GO terms from the categories of biological process (BP), cellular component (CC), and molecular function (MF) were considered significant if they had an adjusted *p*-value < 0.05.

#### 4.5.4. Long Non-Coding RNA (lncRNA) Regulatory Network Construction

BigScale was used for comprehensive analysis of gene interactions across the entire dataset [67]. Z-score transformation was applied to normalize the data and improve the accuracy of correlations between genes. Subsequently, the complex regulatory network was developed to highlight regulatory interactions among specific gene subsets within the network.

## 5. Conclusions

Our study relied on scRNA-seq technology to investigate genome-wide differences in gene expression between non-transformed BEAS2B and transformed H460 lung cancer epithelial cells. BEAS2B cells showed a higher degree of heterogeneity, with gene expression profiles linked to DNA repair and genome integrity, while H460 cells showed deficits in metabolic pathways and uncontrolled proliferation. After carcinogen exposure, BEAS2B cells upregulated metabolic pathways, while H460 cells activated proliferation and wound healing pathways. Our study revealed profound differences in gene expression, particularly in lncRNA expression, including their regulatory networks with protein-coding genes and their interactions with LINE-1 sequences. These findings suggest essential functions of lncRNAs that can help define lung cancer heterogeneity and treatment response.

## Data Availability

Data will be made available on request.

## Supplementary Materials

The following supporting information can be downloaded at https://www.mdpi.com/article/10.3390/ijms26041690/s1.

## Abbreviations

The following abbreviations are used in this manuscript:

NSCLC	Non-small-cell lung cancer
scRNA-seq	Single-cell RNA sequencing
DEGs	Differentially expressed genes
GRN	Gene regulatory network
LncRNA	Long non-coding RNA
LINCRNAs	Long intergenic non-coding RNAs
DMSO	Dimethyl sulfoxide
BaP	Benzo(a)pyrene
QC	Quality control
GO	Gene Ontology
FC	Fold change
TEs	Transposable elements
LINE-1	Long interspersed nuclear element-1
