# Supplementary material for: Single-Cell RNA Sequencing Reveals Extensive Heterogeneity and Unique Gene Trajectories in Non-Transformed and Transformed Human Lung Epithelial Cells: Insights into the Role of LncRNAs in Tumor Heterogeneity
Source: Int J Mol Sci. 2025 Feb 16;26(4):1690. doi: 10.3390/ijms26041690 (PMC11855061; doi:10.3390/ijms26041690)
Supplement: Supplementary file 1 [file ijms-26-01690-s001.zip › ijms-3469463-supplementary.pdf]

International Journal of *Molecular Sciences*

MDPI

*Supplementary Materials*

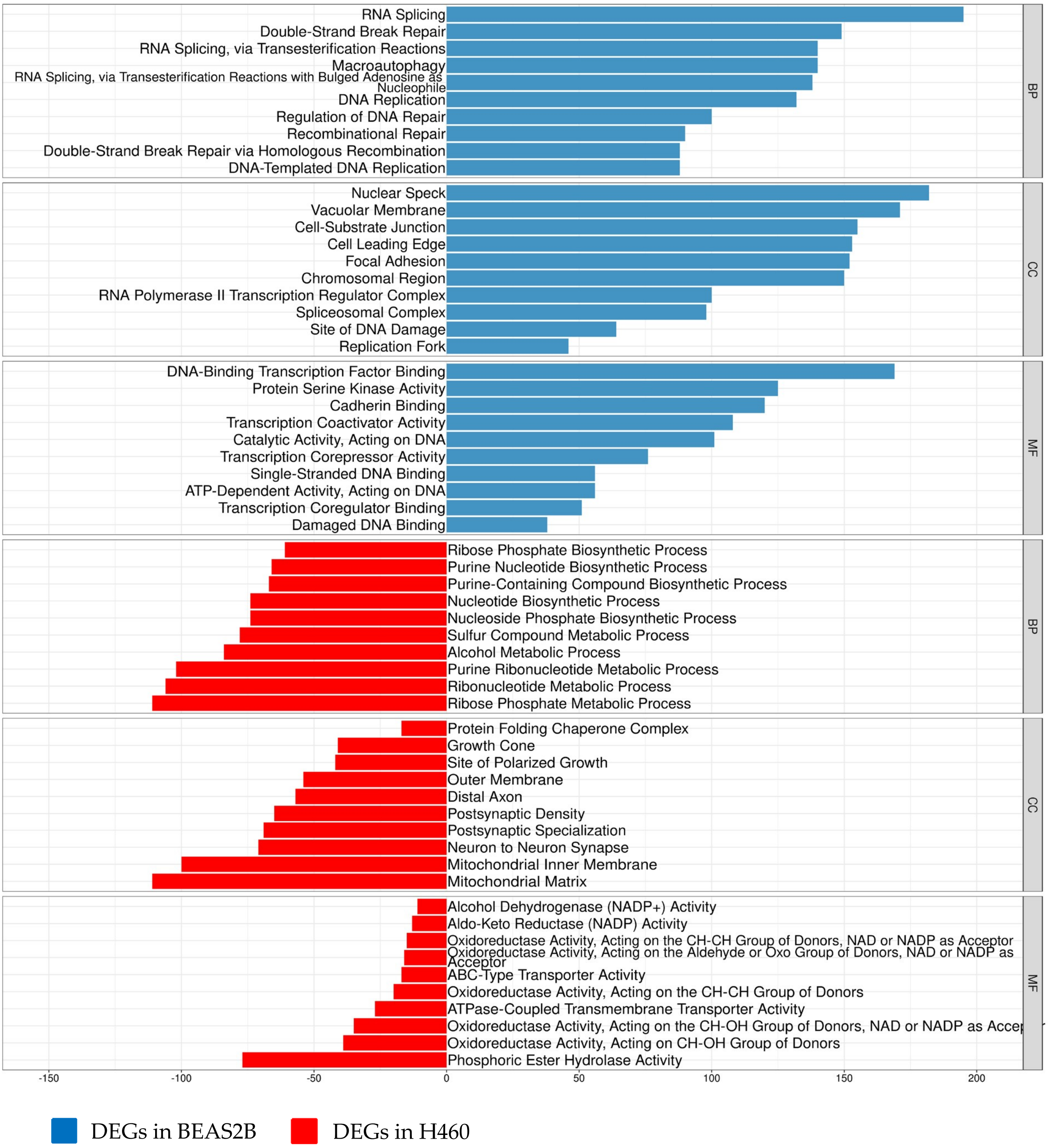


**Figure S1.** Gene Ontology Enrichment Analysis of DEGs in BEAS2B and H460 Cells Under Basal Condition. The top ten terms of biological process (BP), cellular component (CC), and molecular function (MF) with an adjusted p-value $< 0.05$ were identified. The x-axis indicates the number of genes in each category. Blue denotes DEGs in BEAS2B cells and red denotes DEGs in H460 cells.

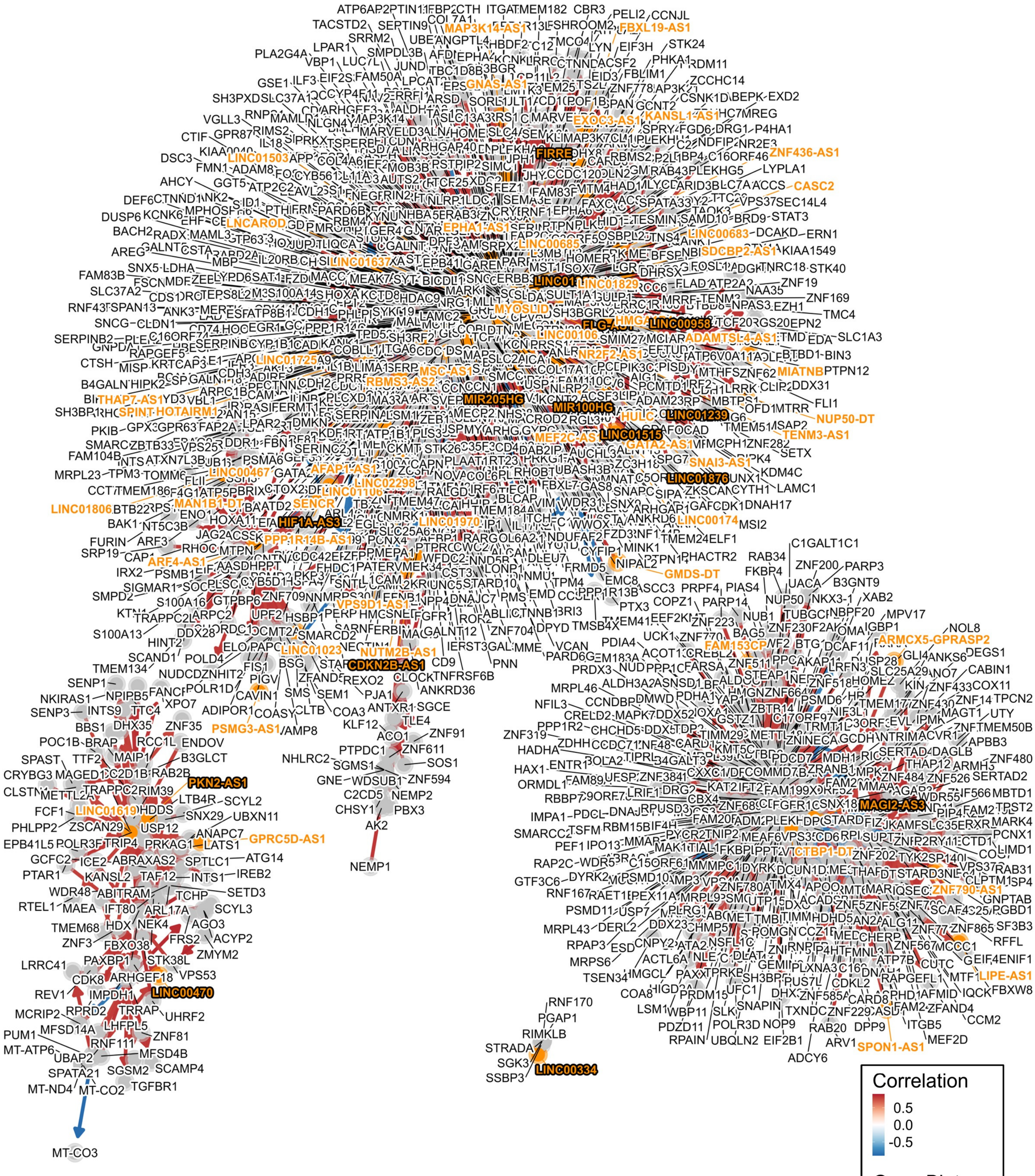


**Figure S2.** Enlarged View of the LncRNA Regulatory Network in BEAS2B Cells Under Basal Condition, as Shown in Figure 2B. Color intensity indicates the degree of correlation, with red denotes positive correlation and blue denotes negative correlation.

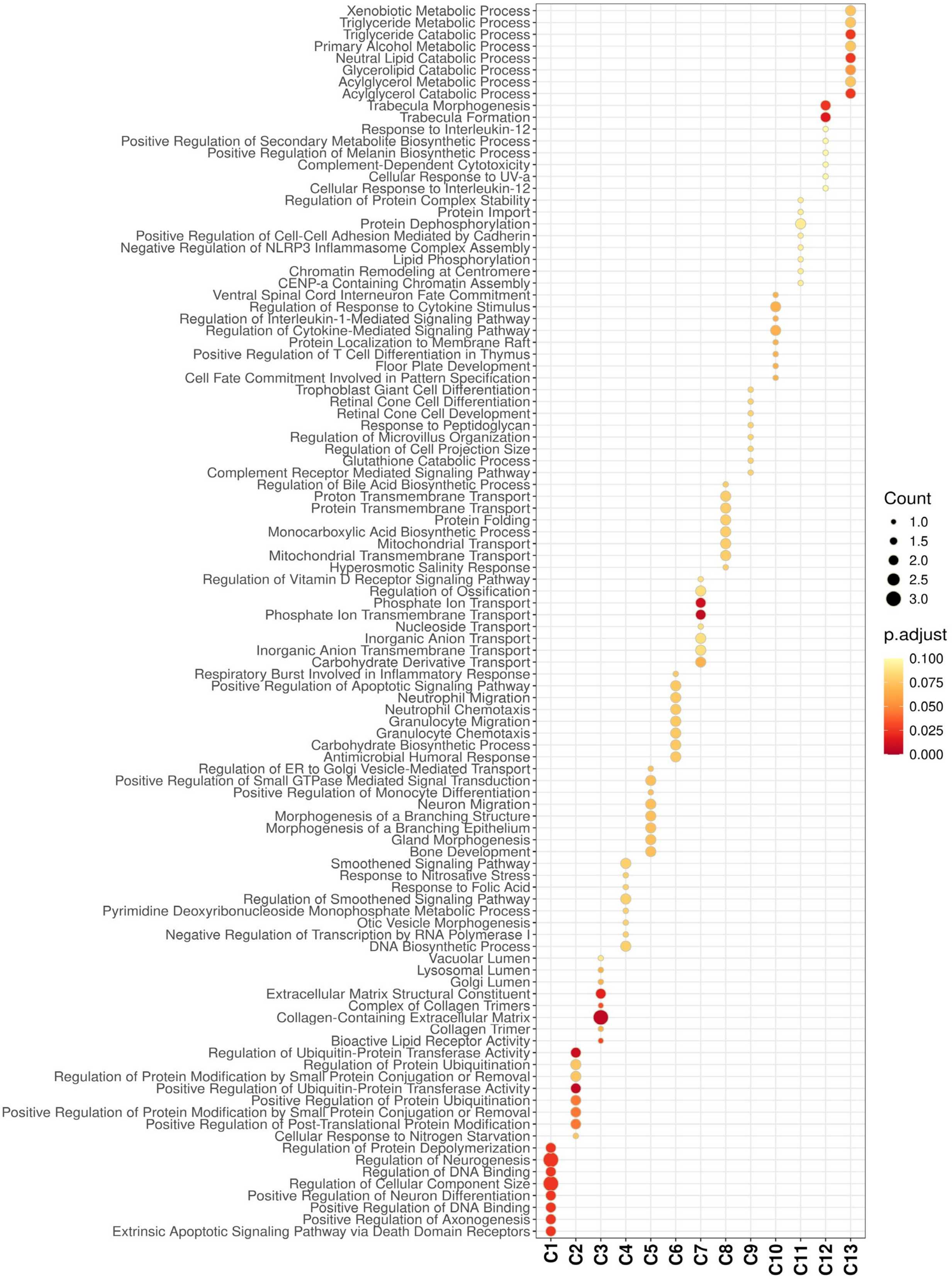


**Figure S3.** Dot Plots of Gene Ontology Enrichment Terms for Specific Subcluster Signatures. The size of the dot represents the number of genes associated with the GO term and the color denotes the p-adjusted values for each term.

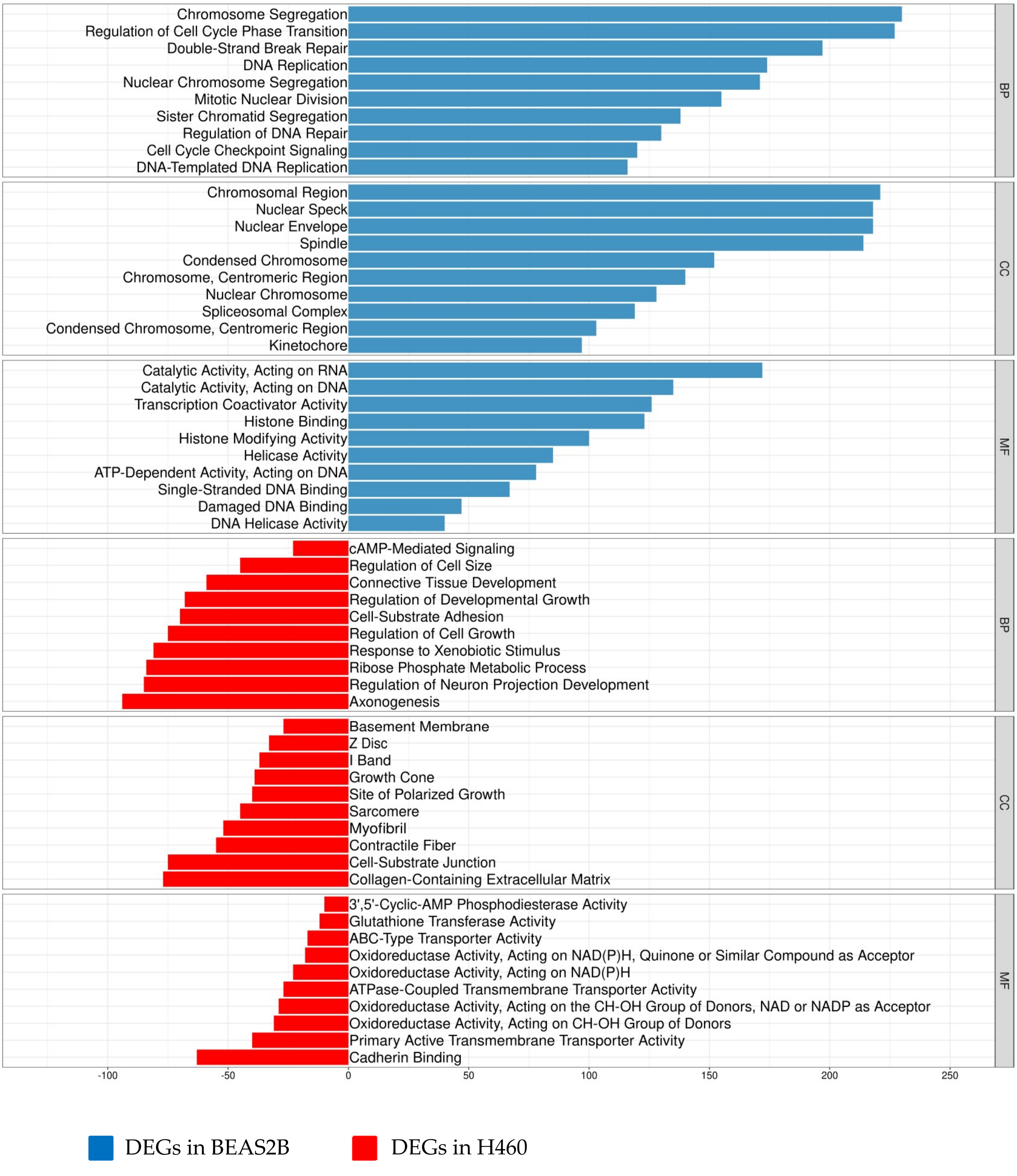


**Figure S4.** Gene Ontology Enrichment Analysis of DEGs in BEAS2B and H460 cells Following Carcinogen Challenge. The top ten terms of biological process (BP), cellular component (CC), and molecular function (MF) with an adjusted p-value < 0.05 were identified. The x-axis indicates the number of genes in each category. Blue denotes DEGs in BEAS2B treated with BaP, while red denotes DEGs in H460 cells treated with BaP.

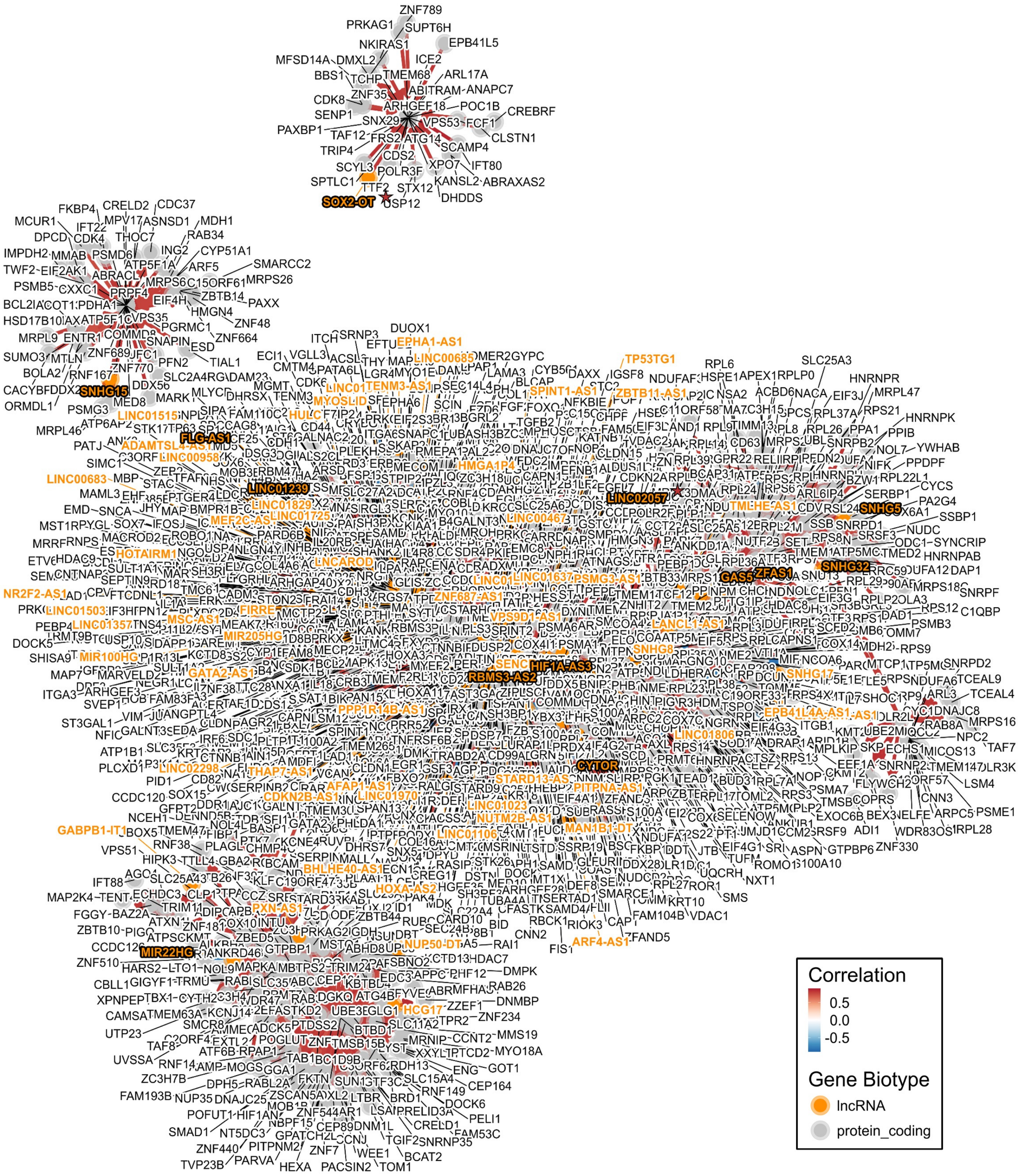


**Figure S5.** Enlarged View of the LncRNA Regulatory Network in DMSO-Treated BEAS2B Cells, as Shown in Figure 6A. Color intensity indicates the degree of correlation, with red denotes positive correlation and blue denotes negative correlation.

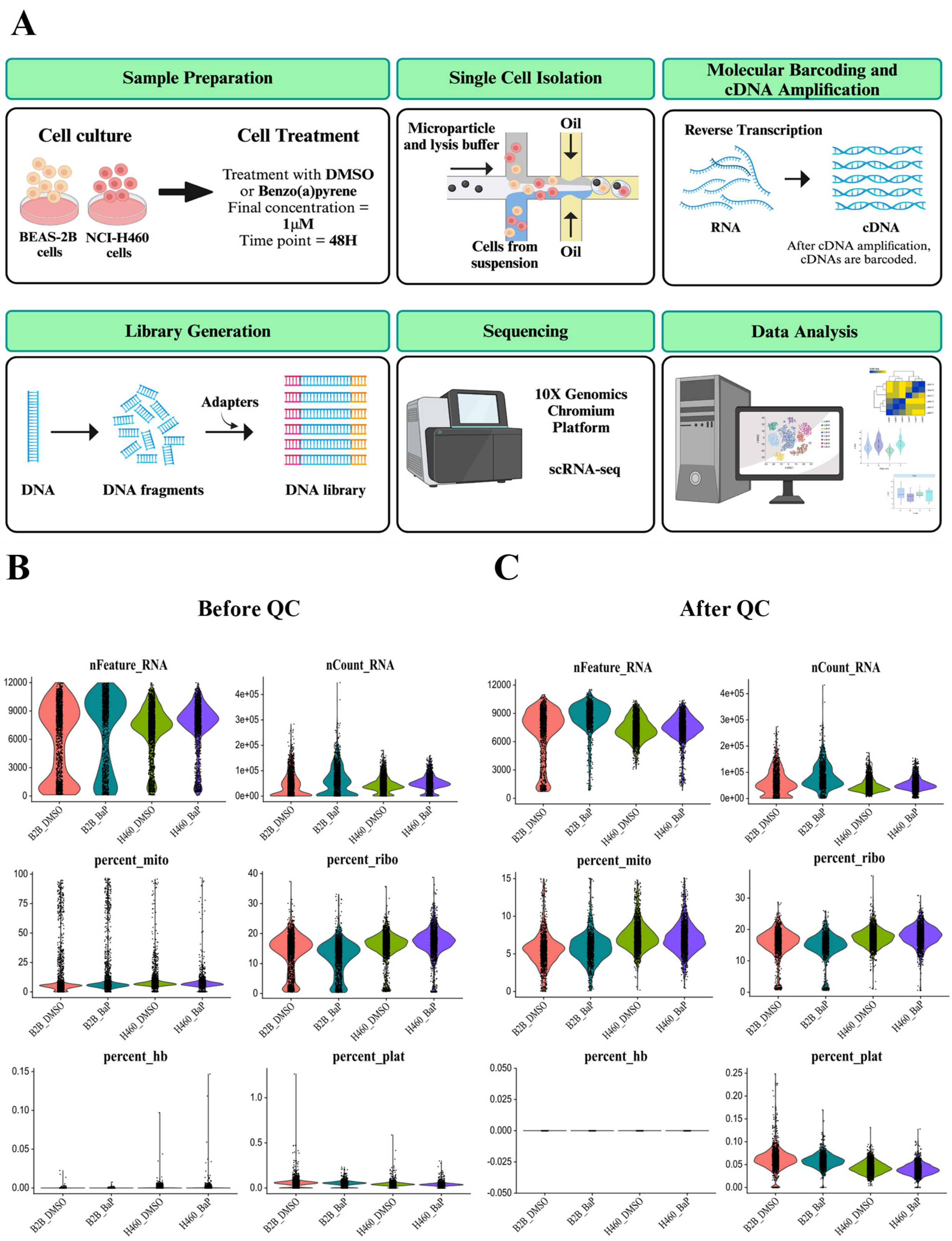


**Figure S6.** Transcriptomic Profiles of Non-transformed BEAS2B and Transformed H460 Lung Epithelial Cells Under Basal and Carcinogen-Stimulated Conditions. (**A**) Schematic representation of the experimental workflow and scRNA-seq process. Isolated single cells and uniquely barcoded beads were captured in droplets using a microfluidics device. mRNAs were reverse transcribed and PCR-amplified to generate a cDNA library. The pooled library was sequenced using a 10X Genomics Chromium platform. Finally, the matrix of single-cell transcriptomes generated was processed for clustering analysis, marker gene identification, and downstream bioinformatics

evaluation. (**B**) Before quality control and (**C**) after quality control, violin plots illustrating the number of genes (nFeature_RNA), number of RNA molecules or counts (nCount_RNA), percentage of mitochondrial genes (percent_mito), percentage of ribosomal RNA genes (percent_ribo), percentage of hemoglobin genes (percent_hb), and percentage of platelet genes (percent_plat) in each cell from the four samples.

**Table S1.** Top 50 differentially expressed protein-coding genes each for BEAS2B and H460 cells with *p*-value < 0.01.

| **BEAS2B cells** | | | | **H460 cells** | | | |
|---|---|---|---|---|---|---|---|
| Gene | Avg $log_2$FC | Gene | Avg $log_2$FC | Gene | Avg $log_2$FC | Gene | Avg $log_2$FC |
| CDKN2A | -12.46 | FBLIM1 | -5.20 | SPP1 | 12.64 | PDE4B | 6.43 |
| MT1E | -11.34 | EPHB2 | -5.39 | EPB41L3 | 12.44 | HOPX | 9.08 |
| CCBE1 | -10.01 | CELF2 | -6.82 | TBXT | 12.16 | HECW1 | 5.77 |
| HMGA2 | -9.37 | TRPS1 | -4.30 | AKR1B10 | 11.57 | GAL | 7.53 |
| PDLIM4 | -10.28 | AKAP12 | -5.86 | GDA | 11.25 | CADM2 | 5.99 |
| ZNF91 | -6.22 | NNMT | -3.25 | PDE10A | 11.64 | PDE1C | 8.37 |
| EYA2 | -6.12 | ITGA3 | -5.29 | GREM2 | 11.25 | GNGT1 | 6.52 |
| SPARC | -8.75 | AXL | -4.66 | DPP10 | 9.74 | ZNF804A | 7.73 |
| CDH4 | -5.58 | EREG | -6.49 | EPHA5 | 9.87 | RTN1 | 7.30 |
| FLI1 | -7.06 | AREG | -5.57 | ALDH3A1 | 10.06 | KIAA0319 | 7.06 |
| KRT17 | -12.31 | ZNF22 | -6.29 | PDE7B | 8.58 | GPAT3 | 6.70 |
| FYN | -4.77 | S100A2 | -7.39 | KCNE4 | 8.64 | LIN7A | 7.46 |
| FBN2 | -4.81 | EHD2 | -5.14 | PDE3B | 8.98 | PDE3A | 7.15 |
| SAA1 | -10.56 | DKK3 | -5.88 | ABCC2 | 7.75 | STXBP6 | 7.42 |
| BICC1 | -5.41 | ZNF334 | -5.31 | AKR1C2 | 8.66 | PELI2 | 5.60 |
| DSP | -6.05 | THBS1 | -6.41 | AKR1C1 | 9.35 | CAVIN2 | 6.51 |
| TPBG | -6.47 | SLC22A15 | -5.44 | EEF1A2 | 7.78 | EPDR1 | 5.75 |
| ZNF625 | -9.36 | SH3RF3 | -4.78 | AKR1C3 | 8.86 | NR0B1 | 7.17 |
| PXDN | -6.23 | MME | -7.78 | ADAM23 | 7.42 | CACNA2D3 | 8.96 |
| ZNF736 | -9.09 | TGFBR2 | -5.14 | ALPK2 | 8.98 | HS3ST3A1 | 8.32 |
| SLC43A3 | -8.86 | CYGB | -5.47 | GPRIN3 | 8.67 | DEFB1 | 10.91 |
| SCRN1 | -5.08 | NAPRT | -4.42 | EBF1 | 9.23 | CLU | 6.38 |
| ZNF682 | -9.28 | BNC1 | -6.56 | NEFL | 9.66 | FHAD1 | 6.27 |
| GSDMD | -6.95 | ZNF331 | -7.30 | S100A4 | 8.47 | TAFA5 | 10.65 |
| RGS17 | -5.42 | THY1 | -8.87 | S100P | 6.31 | CDH11 | 8.07 |

* Avg log2FC < 0 indicates the upregulated protein-coding genes in BEAS2B cells.
* Avg log2FC > 0 indicates the upregulated protein-coding genes in H460 cells.

**Table S2.** Top differentially expressed lncRNA genes in BEAS2B and H460 cells.

| LncRNA gene | *p*-value | Avg $log_2FC$ | LncRNA gene | *p*-value | Avg $log_2FC$ | LncRNA gene | *p*-value | Avg $log_2FC$ |
|---|---|---|---|---|---|---|---|---|
| MicroRNA host genes | | | | | | | | |
| MIR924HG | 1.38E-245 | -1.95 | MIR3936HG | 2.90E-08 | -0.50 | MIR4458HG | 8.87E-82 | 1.83 |
| MIR205HG | 8.94E-226 | -11.15 | MIR3659HG | 8.49E-05 | -2.70 | MIR4500HG | 4.16E-79 | 3.51 |
| MIR100HG | 4.79E-224 | -3.83 | MIR181A2HG | 0.0022 | -0.45 | MIR34AHG | 3.17E-60 | 0.84 |
| MIR31HG | 5.93E-98 | -2.06 | MIR4435-2HG | 0.0331 | -0.10 | MIR762HG | 1.87E-20 | 0.70 |
| MIR503HG | 2.48E-48 | -2.51 | MIR29B2CHG | 0.2579 | -0.56 | MIR17HG | 4.14E-16 | 0.60 |
| MIR181A1HG | 1.21E-27 | -1.04 | MIR4713HG | 0 | 6.46 | MIR1915HG | 4.36E-12 | 0.92 |
| MIR22HG | 1.31E-26 | -0.97 | MIR193BHG | 1.33E-253 | 3.03 | | | |
| MIR222HG | 1.49E-25 | -1.22 | MIR99AHG | 7.85E-107 | 0.69 | | | |
| Antisense RNA genes | | | | | | | | |
| CDKN2B-AS1 | 0 | -9.79 | HIF1A-AS3 | 2.65E-185 | -4.11 | EPHA5-AS1 | 1.11E-258 | 8.44 |
| ZNF433-AS1 | 0 | -5.95 | WARS2-AS1 | 1.42E-174 | -1.95 | DLGAP1-AS1 | 1.51E-240 | 2.75 |
| LURAP1L-AS1 | 0 | -4.28 | PKN2-AS1 | 5.18E-168 | -3.78 | HOXA10-AS | 3.61E-235 | 5.90 |
| MAGI2-AS3 | 1.45E-256 | -4.29 | DPP10-AS1 | 0 | 8.89 | KCNMB2-AS1 | 1.53E-225 | 2.58 |
| FLG-AS1 | 1.23E-207 | -4.80 | DNAH17-AS1 | 0 | 9.09 | BBOX1-AS1 | 1.34E-220 | 4.49 |
| HMGA2-AS1 | 3.94E-199 | -9.49 | ELFN1-AS1 | 6.59E-283 | 7.82 | P3H2-AS1 | 3.40E-214 | 6.48 |
| KIF9-AS1 | 2.89E-186 | -2.16 | PAX8-AS1 | 9.47E-270 | 6.17 | | | |
| Divergent transcript lncRNA genes | | | | | | | | |
| RAB30-DT | 2.49E-88 | -1.16 | NIPBL-DT | 9.89E-08 | -0.51 | MAN1B1-DT | 1.33E-14 | 1.55 |
| BAIAP2-DT | 2.88E-65 | -2.64 | NSMCE1-DT | 0.0002 | -0.43 | RELA-DT | 1.39E-09 | 1.02 |
| LGALSL-DT | 1.64E-47 | -2.17 | LRIG2-DT | 0.0005 | -1.40 | CAPN10-DT | 2.12E-08 | 0.75 |
| ANKRD13C-DT | 3.88E-39 | -1.40 | LYPLAL1-DT | 1.37E-249 | 4.97 | TMED2-DT | 7.22E-08 | 0.97 |
| GMDS-DT | 2.01E-31 | -1.09 | FLVCR1-DT | 3.42E-156 | 3.22 | CTBP1-DT | 9.12E-07 | 0.37 |
| CCDC15-DT | 1.37E-23 | -1.14 | DPH6-DT | 2.75E-89 | 1.15 | ITGB1-DT | 1.33E-05 | 0.45 |
| NUP50-DT | 8.89E-16 | -0.69 | EIF3J-DT | 3.70E-41 | 0.89 | | | |
| Long intergenic non-protein coding RNA genes | | | | | | | | |
| LINC00470 | 0 | -7.43 | LINC01239 | 8.06E-162 | -5.59 | LINC00922 | 0 | 7.81 |
| LINC00958 | 0 | -8.89 | LINC00882 | 6.63E-160 | -3.46 | LINC00355 | 0 | 8.16 |
| LINC00511 | 0 | -4.85 | LINC01357 | 2.26E-119 | -5.07 | LINC01341 | 4.42E-300 | 4.49 |
| LINC01876 | 2.44E-243 | -8.34 | LINC01035 | 0 | 11.87 | LINC01515 | 9.09E-251 | 1.96 |
| LINC00886 | 1.39E-220 | -3.73 | LINC00824 | 0 | 12.75 | LINC02320 | 4.30E-232 | 7.33 |
| LINC00334 | 1.51E-195 | -5.09 | LINC01508 | 0 | 8.39 | LINC00632 | 8.55E-221 | 3.88 |
| LINC00571 | 1.76E-163 | -4.00 | LINC00942 | 0 | 9.03 | | | |
| Other lncRNA genes | | | | | | | | |
| SFTA1P | 0 | -10.81 | PCAT1 | 2.18E-235 | -4.25 | CRNDE | 0 | 7.98 |
| CASC19 | 0 | -9.94 | SNHG17 | 1.34E-215 | -1.46 | LUCAT1 | 0 | 4.74 |
| ZFAS1 | 0 | -2.14 | FAM106A | 7.86E-193 | -6.31 | DLEU1 | 0 | 2.48 |
| MALAT1 | 0 | -1.69 | FIRRE | 0 | 7.61 | SNHG19 | 0 | 2.40 |
| GAS5 | 1.06E-307 | -1.43 | CASC9 | 0 | 9.60 | PVT1 | 0 | 1.47 |
| SNHG12 | 7.81E-295 | -3.47 | DUXAP8 | 0 | 6.65 | DANT2 | 1.43E-227 | 1.14 |
| SNHG32 | 2.18E-276 | -1.94 | PART1 | 0 | 5.62 | | | |

* Avg log2FC < 0 indicates the upregulated lncRNA genes in BEAS2B cells.

* Avg log2FC > 0 indicates the upregulated lncRNA genes in H460 cells.

**Table S3.** The number of cells in each subcluster.

| Main Treatment Group | Subclusters | Size (Number of Cells) |
| --- | --- | --- |
| BEAS2B-DMSO | C1 | 780 |
| | C2 | 575 |
| | C3 | 115 |
| BEAS2B-BaP | C4 | 283 |
| | C5 | 132 |
| | C6 | 100 |
| | C7 | 187 |
| H460-DMSO | C8 | 535 |
| | C9 | 911 |
| | C10 | 561 |
| H460-BaP | C11 | 704 |
| | C12 | 734 |
| | C13 | 193 |

**Table S4.** Top 50 differentially expressed protein-coding genes for DMSO- and BaP- treated BEAS2B cells.

| DMSO-treated group | | | BaP-treated group | | |
| --- | --- | --- | --- | --- | --- |
| Gene | *p*-value | Avg $log_2FC$ | Gene | *p*-value | Avg $log_2FC$ |
| RPS29 | 8.05E-160 | -0.74 | DHRS3 | 2.13E-172 | 3.21 |
| RPS21 | 2.37E-140 | -0.51 | FDPS | 6.10E-164 | 0.97 |
| RPL37A | 4.59E-114 | -0.49 | TKT | 2.38E-139 | 1.01 |
| TXNIP | 8.92E-103 | -1.79 | FDFT1 | 2.22E-126 | 1.23 |
| RPL38 | 4.55E-91 | -0.46 | CYP51A1 | 1.29E-122 | 0.80 |
| ATP5F1E | 9.24E-79 | -0.37 | NQO1 | 2.11E-121 | 1.17 |
| RPL36A | 4.40E-76 | -0.43 | CYP1B1 | 1.48E-118 | 1.49 |
| RPL36 | 1.38E-73 | -0.42 | SERPINB1 | 2.25E-116 | 1.19 |
| MT-ND3 | 6.23E-68 | -0.44 | IGFBP6 | 2.60E-115 | 1.46 |
| SPIRE1 | 1.67E-65 | -0.72 | ACAT2 | 6.98E-108 | 0.85 |
| KTN1 | 3.75E-65 | -0.35 | SCD | 6.84E-99 | 0.96 |
| ARHGAP26 | 9.30E-59 | -0.85 | G6PD | 4.92E-96 | 0.94 |
| RPL37 | 3.93E-58 | -0.41 | TIPARP | 9.27E-85 | 0.94 |
| ARHGEF28 | 3.42E-57 | -0.73 | DHCR7 | 1.38E-84 | 1.07 |
| RPL14 | 9.15E-56 | -0.31 | LGALS3BP | 3.42E-83 | 0.76 |
| RPL12 | 6.24E-55 | -0.31 | DHCR24 | 3.95E-78 | 0.76 |
| RPS28 | 7.05E-55 | -0.35 | LTBP3 | 2.59E-77 | 1.48 |
| PAWR | 1.21E-53 | -0.51 | HPCAL1 | 2.13E-76 | 0.99 |
| COL8A1 | 1.48E-53 | -1.50 | RPL7 | 2.53E-76 | 0.57 |
| RPL22L1 | 2.72E-52 | -0.80 | TXNRD1 | 5.86E-76 | 0.75 |
| CCN1 | 6.18E-52 | -0.97 | MED24 | 1.46E-74 | 1.20 |
| EIF3J | 1.22E-50 | -0.50 | FADS2 | 4.01E-74 | 0.81 |
| TMA7 | 1.81E-49 | -0.38 | ETFB | 5.02E-74 | 0.72 |
| RPS27 | 3.43E-47 | -0.33 | EBP | 3.47E-73 | 0.65 |
| CDH4 | 4.02E-46 | -1.37 | PCYT2 | 5.77E-73 | 1.26 |

* Avg log2FC > 0 indicates the upregulated protein-coding genes upon BaP treatment.
* Avg log2FC < 0 indicates the downregulated protein-coding genes upon BaP treatment.

**Table S5.** Top 50 differentially expressed protein-coding genes for DMSO- and BaP- treated H460 cells.

| **DMSO-treated group** | | | **BaP-treated group** | | |
|---|---|---|---|---|---|
| Gene | *p*-value | Avg $log_2FC$ | Gene | *p*-value | Avg $log_2FC$ |
| TRIB3 | 0 | -2.57 | BTG2 | 0 | 4.19 |
| MCM7 | 0 | -1.87 | SULF2 | 0 | 4.76 |
| PYCR1 | 0 | -1.96 | CDKN1A | 0 | 3.81 |
| PHGDH | 0 | -1.73 | ACTA2 | 0 | 3.17 |
| SLC1A5 | 0 | -1.69 | CYFIP2 | 0 | 2.95 |
| SLC7A5 | 0 | -1.72 | ZMAT3 | 0 | 2.63 |
| RPL22L1 | 0 | -2.85 | PHLDA3 | 0 | 2.65 |
| PSAT1 | 0 | -1.65 | FDXR | 0 | 3.17 |
| EIF4EBP1 | 0 | -1.49 | GDF15 | 0 | 3.39 |
| FASN | 0 | -1.61 | TIGAR | 0 | 2.10 |
| HMGN2 | 0 | -1.45 | TP53I3 | 0 | 4.08 |
| SCD | 0 | -1.86 | BAX | 0 | 1.73 |
| MTHFD2 | 0 | -1.34 | MDM2 | 0 | 3.99 |
| ATF4 | 0 | -1.58 | ISCU | 0 | 1.43 |
| FADS1 | 0 | -1.64 | RPS27L | 0 | 2.24 |
| MRPL12 | 0 | -1.34 | NSD1 | 0 | 1.08 |
| DEK | 0 | -1.17 | BTF3 | 0 | 0.73 |
| EIF3J | 0 | -1.23 | PHPT1 | 0 | 0.99 |
| ATP5ME | 0 | -1.41 | ALDH3A1 | 0 | 2.84 |
| MT-ND4L | 0 | -1.26 | S100A11 | 0 | 0.78 |
| HNRNPAB | 0 | -1.10 | MYOF | 0 | 1.21 |
| PGD | 0 | -0.88 | S100A10 | 0 | 1.47 |
| ANP32B | 0 | -0.80 | RPS10 | 0 | 0.86 |
| VDAC1 | 0 | -0.82 | RPS4X | 0 | 0.66 |
| ANKRD11 | 0 | -0.89 | HINT1 | 0 | 0.62 |

* Avg log2FC > 0 indicates the upregulated protein-coding genes upon BaP treatment.
* Avg log2FC < 0 indicates the downregulated protein-coding genes upon BaP treatment.

**Table S6.** Full-length LINE-1 elements located within DMSO- and BaP-induced lncRNAs in BEAS2B and H460 cell lines.

| LncRNAs | LINE-1 Retrotransposons | Genomic Size of LINE-1 (bp) | Position of LINE-1 |
|---|---|---|---|
| BEAS2B-DMSO | | | |
| LINC01505 | L1PA2 | 6053 | chr9:106181589-106187641 |
| LINC01505 | L1PA7 | 6140 | chr9:106198352-106204491 |
| LINC01505 | L1PA5 | 6142 | chr9:106604989-106611130 |
| LINC02057 | L1PA4 | 6127 | chr5:61238875-61245001 |
| ZFPM2-AS1 | L1PA3 | 6145 | chr8:105789090-105795234 |
| ZFPM2-AS1 | L1MA1 | 6096 | chr8:105815923-105822018 |
| ZFPM2-AS1 | L1PA5 | 6106 | chr8:106023329-106029434 |
| SOX2-OT | L1PA8A | 6442 | chr3:181069351-181075792 |
| SOX2-OT | L1PB1 | 6966 | chr3:181223939-181230904 |
| SOX2-OT | L1PA7 | 6176 | chr3:181319257-181325432 |
| SOX2-OT | L1PA5 | 6161 | chr3:181585392-181591552 |
| BEAS2B-BaP | | | |
| RMDN2-AS1 | L1PA4 | 6163 | chr2:38004696-38010858 |
| RMDN2-AS1 | L1PA7 | 6509 | chr2:37956657-37963165 |
| OLMALINC | L1PA4 | 6170 | chr10:100406331-100412500 |
| EIF1B-AS1 | L1PA3 | 6039 | chr3:40154991-40161029 |
| EIF1B-AS1 | L1PA5 | 6118 | chr3:40271391-40277508 |
| H460-DMSO | | | |
| OLMALINC | L1PA4 | 6170 | chr10:100406331-100412500 |
| LINC00662 | L1PA4 | 6149 | chr19:27695439-27701587 |
| LINC01505 | L1PA2 | 6053 | chr9:106181589-106187641 |
| LINC01505 | L1PA7 | 6140 | chr9:106198352-106204491 |
| LINC01505 | L1PA5 | 6142 | chr9:106604989-106611130 |
| ENTPD1-AS1 | L1PA7 | 6440 | chr10:95928411-95934850 |
| H460-BaP | | | |
| RMDN2-AS1 | L1PA4 | 6163 | chr2:38004696-38010858 |
| RMDN2-AS1 | L1PA7 | 6509 | chr2:37956657-37963165 |
| LINC00472 | L1PA7 | 6427 | chr6:71355362-71361788 |
| LINC02328 | L1PA2 | 6035 | chr14:86105460-86111494 |
| LINC02328 | L1PA3 | 6028 | chr14:86074490-86080517 |
| LINC02328 | L1PA3 | 6107 | chr14:86095935-86102041 |
| LINC02328 | L1PA6 | 6161 | chr14:85969036-85975196 |
| LINC02328 | L1PA7 | 6414 | chr14:86042353-86048766 |
| CYP1B1-AS1 | L1PA4 | 6185 | chr2:38132408-38138592 |
| LINC02057 | L1PA4 | 6127 | chr5:61238875-61245001 |

**Table S7.** High sequencing quality for all samples.

| | BEAS2B-DMSO | BEAS2B-BaP | H460-DMSO | H460-BaP |
|---|---|---|---|---|
| Sequencing | | | | |
| Number of Reads | 192,898,745 | 218,649,039 | 213,490,031 | 188,103,665 |
| Valid Barcodes | 97.4% | 96.9% | 95.1% | 96.9% |
| Valid UMIs | 100.0% | 99.9% | 100.0% | 100.0% |
| Mapping | | | | |
| Reads Mapped to Genome | 93.2% | 92.3% | 91.5% | 93.5% |
| Reads Mapped Confidently to Genome | 89.3% | 88.0% | 87.4% | 90.3% |
| Reads Mapped Confidently to Intergenic Regions | 4.3% | 4.2% | 5.1% | 6.0% |
| Reads Mapped Confidently to Intronic Regions | 24.2% | 24.9% | 24.3% | 26.6% |
| Reads Mapped Confidently to Exonic Regions | 60.9% | 59.0% | 57.9% | 57.8% |
| Reads Mapped Confidently to Transcriptome | 75.9% | 74.5% | 74.0% | 76.7% |
| Reads Mapped Antisense to Gene | 8.4% | 8.7% | 7.6% | 6.9% |
| Cells | | | | |
| Estimated Number of Cells | 1,803 | 1,427 | 2,390 | 1,842 |
| Fraction Reads in Cells | 82.3% | 83.7% | 91.9% | 92.7% |
| Mean Reads per Cell | 106,988 | 153,223 | 89,326 | 102,119 |
| Median UMI Counts per Cell | 34,966 | 52,373 | 39,656 | 49,644 |
| Median Genes per Cell | 7,284 | 8,713 | 7,595 | 8,078 |
| Total Genes Detected | 28,072 | 27,973 | 28,811 | 28,461 |

* *Sequencing*: Numbers of sequencing reads.
* *Mapping*: Mapping of sequencing reads to the human genome.
* *Cells*: Distribution of sequencing reads in single cells and numbers of cells with valid reads.
